# Clinical Work and Life of Mid-Career Male Nurses: A Qualitative Study

**DOI:** 10.3390/ijerph18126224

**Published:** 2021-06-08

**Authors:** Soo-Yong Shin, Eun-Ju Lim

**Affiliations:** 1 Seoul Metropolitan Government-Seoul National University Boramae Medical Center, 20 Boramae-ro 5-gil, Dongjak-gu, Seoul 07061, Korea; kmssy7078@gmail.com; 2 Red Cross College of Nursing, Chung-Ang University, 84 Heukseok-ro, Dongjak-gu, Seoul 06974, Korea

**Keywords:** Korea, nursing, male, qualitative research, workplace

## Abstract

In Korea, about 3000 qualified male nurses enter the clinical nursing field annually; however, they face challenges in long-term job retention in general hospitals. Therefore, this study characterized the work and life experiences of mid-career male nurses engaged in clinical nursing care. Participants were nine registered nurses with a minimum of five years’ work experience in a general hospital in Korea. Data were collected through face-to-face, in-depth, semi-structured interviews. A phenomenological qualitative design was used with Colaizzi’s data analysis method. Three categories, eight theme clusters, and seventeen themes were revealed. The three categories were “limitations and adaptation to work performance”, “interpersonal difficulties and coping”, and “facing reality and preparing for the future”. Findings suggested that mid-career male nurses were considering transitioning to other job roles. These findings help clarify the clinical work experience of mid-career male nurses and their difficulties with job retention. The results provide basic data that may inform the design of policies to practically support male nurses in preserving their careers.

## 1. Introduction

The proportion of registered male nurses is 23% in the Netherlands, 11.7% in Australia, 11% in the UK, 9.6% in the US, and 9% in New Zealand [1]. The number of male nurses in Korea increased from approximately 2000 in the early 2000s to 11,880 in 2017, which accounted for 3.3% of all 360,000 nurses [2]. The proportion of male nursing students also increased rapidly from 4.7% in 2004 to approximately 15% in 2017 [3]. This increase in the number of male nurses could be attributed to changing social norms that led to gender stereotypes being less pervasive and allowed individuals to select jobs that best suit their aptitude, and to the increased flexibility of members of the younger generation in terms of their job perceptions [4]. In addition, important factors that influence men’s decision to enter nursing include the ease of obtaining a nursing job [5], the relatively high salary drawn as a new employee [6], and the anticipation of faster achievement in a field in which men are a minority [7,8].

Although the number of male nurses is increasing, men remain the minority group in nursing [9]. In many countries, nursing is still a women-led profession, and is sometimes perceived as a “woman’s job” [9,10]. In general, it is believed that women tend to internalize feminine work abilities, such as caring for or helping others, while men tend to internalize masculine work abilities such as initiative and management systems [11,12]. In the nursing profession, gender, race, and class are factors that affect the ability of individuals to get higher wages and advance to senior positions on the glass escalator, which is reported to be advantageous for male nurses [9,13]. A recent study in the US reported a difference between the earnings of male and female registered nurses to be as high as USD 10,000 [14].

Gender stereotypes continue to exist owing to conservative views and the social climate, which has led to difficulties for male nurses in adapting to the clinical workforce and has increased their job stress. In turn, these factors affect male nurses’ career management [15,16]. Male nurses tend to face problems with stereotypes that can hinder their focus on nursing [17]. They experience workplace discrimination in employment opportunities or promotion, quit their jobs, or move to special fields such as psychiatry, emergency rooms, operating rooms, or intensive care units to escape the “feminine image” of nursing [10]. The unequal distribution of women and men within the profession can lead to negative consequences such as gender wage gaps in the labor market, as well as gender inequality in the workplace environment. In Korea, some patients are not used to receiving care from male nurses and they would rather form and maintain working relationships with female nurses, who are more numerous [18]. Additionally, novice male nurses are subjected to a rigid, military-like, organizational culture by senior male nurses [7]. Further, married male nurses have reported being worried about not only infrequent pay raises, and physical and mental exhaustion from continuous shift work, but also a lack of family stability [19]. Therefore, approximately 36% of male nurses change jobs, while others plan to change their job in the future [19]. Some male nurses recognize their nursing career as a bridge to move into other fields [4], highlighting the lack of a long-term commitment to nursing [20].

The clinical practice experience of mid-career male nurses likely differs from that of female or recently-graduated nurses; to date, the latter two groups have been the main focus of research in this field [7,21]. The present study aimed to clarify the working reality of mid-career male nurses by depicting their work experiences. In addition, it aimed to provide basic data to help devise a working environment and organizational culture that fosters male clinical nurses’ long-term job retention.

## 2. Materials and Methods

### 2.1. Design

This phenomenological qualitative study was conducted to clarify the lived experiences of mid-career male nurses in clinical practice.

### 2.2. Participants

One of the authors is a male nurse and a member of the Korean Male Nursing Association. With the permission of the association’s committee, the researcher used the website of the Korean Male Nursing Association to explain the research topic, purpose, and criteria for selecting participants, and made an announcement for recruiting participants. In this study, a mid-career nurse was defined as a staff nurse with 5–15 years of experience of working as a registered nurse in a general hospital. This is based on previous research that shows that the experience of experienced workers is different from that of new male nurses who have just joined after graduation [6]. Subsequently, interviews were conducted with those participants who expressed their wish to voluntarily participate in the study via a contact form. Finally, nine male mid-career nurses were recruited using purposive sampling.

### 2.3. Data Collection

Data were collected from March to November 2017 through face-to-face, in-depth observation, evidence documentation, audio recording, transcription, semi-structured interviews, and note-taking during the interviews. Subsequently, interview content was confirmed, and additional questions were posed via methods such as face-to-face interviews, phone calls, or e-mails for a maximum of three sessions. The researchers collected various types of data so that they could more accurately analyze the participants. The length of the interviews varied between one and two hours. The questions began with, “Could you tell me about your experience with working in the hospital as an experienced male nurse?” followed by open-ended questions, such as “What are some things you worry about as an experienced male nurse?” Where necessary, we asked more in-depth questions, such as “Could you tell us more details about this content?” and “What was your strategy for adjusting to life in the ward?”. We wanted to capture participants’ experiences as much as possible through questions from various perspectives, such as “What was the change in the relationship between medical staff and administrative staff, including nurses?”, “What does it mean to you to be a husband and a father with a nurse’s job?”, and “What is your future as a nurse?”. With such questions, we expanded it to the realm of life. In the process of answering, data were collected through questions, such as “How did you feel in that situation?” and “Can you tell me more about the content?”, when researchers wanted to know the situation a little more or when it was judged that specific answers were needed. The researchers showed that they were concentrating by observing the participant’s unconscious behavior and expression during the interview and responding to the participant’s words. In addition, during the interview, the participants’ behavior, facial expressions, unconscious behavior, and use of a specific tone were carefully observed and noted onsite. Interview transcripts were printed in 11-point font on A4 size paper (N = 135 sheets). The interviews proceeded until data saturation was achieved, that is, until no new data were discovered [22]. Data saturation was achieved at mid-career male nurse participant number eight. One more participant was interviewed, and no new information emerged.

### 2.4. Ethical Considerations

This study was approved by the ethics board of the first author’s institution of affiliation (approval no. 1041078-201702-HR-030-01). All participants received information about the study, and they provided written consent before participating.

### 2.5. Data Analysis

Data were transcribed verbatim and analyzed using Colaizzi’s [23] method. This approach provides a clear and structured process for analyzing data. This six-step process includes (i) listening to the recorded interview content, observing the imprinted content, and understanding the emotions associated with the situation and their approximate meaning; (ii) extracting meaningful sentences or phrases related to participants’ experience; (iii) giving meaning to general and abstract statements; (iv) carefully checking and analyzing whether themes, theme clusters, and categories match the meaning of the source data; (v) collectively describing the data analyzed according to the theme, the theme cluster, and the category; and (vi) finalizing the analysis and producing the report. Data analysis was performed concurrently while data were collected. Comprehensive documentation was generated throughout the project, ensuring the existence of an audit trail.

### 2.6. Trustworthiness

Assessment of strictness is the last step in determining the quality of research in qualitative studies. The researchers assessed truth value, applicability, consistency, and neutrality as per the evaluation standards recommended by Lincoln and Guba [24]. To determine truth value, the researchers showed the interview records and analyses to all nine participants, who confirmed whether the contents were consistent with their experiences. This included peer review, which allows for more objective analysis by considering the different interpretations of researchers. To determine applicability, the researchers also showed the analysis material to four mid-career male nurses who did not participate in the study and sought their opinion on whether the content could be applied as meaningful research material, based on their own experience. To determine consistency, the researchers followed Colaizzi’s method throughout data collection and analyses, and received confirmation from two professors with experience in phenomenology, who also provided guidance concerning the qualitative research. To determine neutrality, prejudices were recorded in a personal diary and the researchers strived to discard any personal biases.

## 3. Results

### 3.1. Participants’ Demographic Characteristics

Participants’ demographic characteristics are shown in Table 1. Nine male participants provided information about their clinical work experience (age range = 30–38 years). They all worked in general hospitals in Seoul, Korea at the time of the study. Of the nine participants, four worked in an emergency room, two in an operating room, one in a psychiatric ward, one in an internal medicine ward, and one at an outpatient cardiac center. Their individual clinical work experience ranged from 5–13 years. The thematic analysis is shown in Table 2.

### 3.2. Category 1. Limitations and Adaptation to Work Performance

#### 3.2.1. Theme Cluster 1. Compliance with the Workplace Culture

This cluster consisted of the themes “living in a vertical ward culture” and “paying your dues”. Participants felt that their working environment was rigid and that their hierarchy was similar to a strict vertical military culture. However, rather than trying to change it themselves, they adhered to the ward culture.

“I think hospitals are a social version of the military. I do think that the excessively strict hierarchical work environment and system based on the years of employment or rank are unreasonable and unproductive; but that doesn’t change easily.”

(Participant 8)

Participants said they had to strive to adjust to their department and that having to overcome extreme situations helped them perform their jobs more easily than before.

“As I continued to work without an accident or a mishap, the preceptors and senior nurses must have thought that I would work here for a long time and started treating me differently. This means that, in order to settle in a new place, I need to work really hard.”

(Participant 1)

#### 3.2.2. Theme Cluster 2. Being Recognized as a Member of the Organization

This cluster consisted of the themes “adapting to a traditionally female environment” and “working smoothly”. Although participants gained experience within the department, they remained the minority sex. Some participants felt more scrutinized for minor mistakes or specific comments they made.

“I don’t talk about personal matters other than work. I think it’s a gender-specific cultural difference, but it seems that they distort my meaning rather than listen to what I’m saying. There are miscommunications that can arise from that …”

(Participant 3)

They also described themselves as tending to show passive and defensive behavior while trying to determine the mood of senior and female nurses.

“I don’t want to cause trouble by acting out and trying to solve problems myself. I don’t even know that the more junior nurses are ready to accept and fix their behavior based on what I teach anyway. I worry that I would unnecessarily worsen the culture of the department.”

(Participant 1)

#### 3.2.3. Theme Cluster 3. New Role Burden

This cluster included the themes “new work expectations” and “taking initiative”. As nurses gained experience, they were assigned several jobs, such as leading a teaching group and running seminars within the department. Although participants felt burdened, they expended the effort required to achieve optimal results.

“I became the charge nurse of the orthopedics surgery room. The Head Nurse gave me the job of caring for and teaching junior nurses. I am trying to make them A-grade practitioners by strictly educating and sometimes disciplining them.”

(Participant 4)

#### 3.2.4. Theme Cluster 4. Physical Exhaustion

This cluster consisted of the themes “lack of sleep and chronic fatigue” and “burden of night shifts and a lack of physical fitness”. Participants complained of chronic exhaustion and lethargy due to continual shift work. They experienced a decline in physical strength with each passing year.

“Definitely with shift work, I can’t sleep well, and [my] life pattern is irregular. The exhaustion just does not end.”

(Participant 3)

“For a few years in the beginning, my physical strength recovered quickly even if I worked night shift. Now that I’m in my late 30s, the recovery speed is getting slower.”

(Participant 6)

### 3.3. Category 2. Interpersonal Difficulties and Coping

#### 3.3.1. Theme Cluster 1. Strategy and Support for Survival

This cluster consisted of the themes “communicating with each other”, “support of male colleagues”, and “encouragement received from fellow and senior nurses”. Participants thought that communicating with each other when working at a ward was necessary to prevent miscommunication and to maintain relationships with co-workers.

“I think that when I get criticized for a mistake, admitting the mistake, putting in the effort to not make the same mistake, controlling my facial expressions, and communicating with manners to wrap it up are the most important.”

(Participant 8)

Participants often communicated with male paramedics and office workers in other departments when there were no male nurses within their own department.

“When I first got assigned the role in the department in 2004, I was the only male out of 35 nurses. No matter how close I got with female nurses I couldn’t ignore the differences in thought that stem from gender. People that were there for me then were the staff and paramedics.”

(Participant 1)

Teamwork and harmony with fellow nurses aided participants’ adaptation. They relied on advice from senior nurses. In addition, participants whose spouses were also nurses were thankful that they empathized with the hardships of hospital life and encouraged them to continue to work in this field.

“Every time, the Head Nurse gives a lot of advice on what the best way is to do things in hospital life. I think I came to rely on that a lot.”

(Participant 7)

#### 3.3.2. Theme Cluster 2. Improved Patient Relationships

This cluster consisted of the themes “easier clinical practice than ever before” and “positive perception toward male nurses”. Participants said that, compared to when they were hired, they were now more adept at handling patients and that patients treated them better.

“Definitely, with more experience, better work techniques and skills are obvious. But this is also important in the relationship with patients. I think that there is more trust in the relationship with patients because of these things.”

(Participant 8)

Participants also reported that patients who visited frequently considered them trustworthy rather than unfamiliar. They felt that these patients believed that having a male nurse had become more common.

“When I first got hired and I told patients during rounds that I was a nurse, most patients responded like, “A man?”. Now, maybe because there are a few male nurses in the department and male nurses are more common in channels of mass communication, patients who visit often show me that they know me and treat me kindly.”

(Participant 5)

### 3.4. Category 3. Facing Reality and Preparing for the Future

#### 3.4.1. Theme Cluster 1. Growth Limitations

This cluster consisted of the themes “familial financial burden” and “lack of role models”. Although most participants had a stable work life with a comparatively high salary, they were skeptical about promotion and questioned how long they would be able to work at the hospital without promotion. All of them had families to support, which was an additional source of pressure.

“I worked here for a long time and the salary is okay. But with more age and growing children, how many people would be willing to work as a regular nurse to work until retirement? At least if I become the Head Nurse I can be guaranteed my future until retirement; but that is not easy.”

(Participant 1)

Another reason for anxiety about the future was that participants could not observe a successful senior male nurse as a role model.

“From what I can tell there are almost no male nurses from the beginning that hold high positions. What good is an increase in male nursing students? Most quit after a little while and I think the lack of a definite role model may be one of the reasons. This is why male nurses need to be pioneers in the field of nursing.”

(Participant 2)

#### 3.4.2. Theme Cluster 2. Preparing for a Better Future

This cluster consisted of the themes “developing competence through self-reflection” and “considering changing job roles”. Participants said that they developed a strength or an interest while working, and that they were using this for self-development and to plan a different future.

“When I linked my interests to my studies, it connected to insurance evaluation jobs. I had a thought that it would be good to go into an insurance evaluation team when given a chance, and I am currently preparing to obtain a certificate for it.”

(Participant 4)

However, some participants experienced uncertainty when they observed other male nurses leave the nursing profession and when they heard stories about those who had already changed jobs. Some participants began to develop an interest in other job roles, and they considered changing jobs.

“All four male nursing students in my college cohort quit hospitals to become government firefighters, and most male juniors and seniors from when I was in college also became government firefighters. Hearing that, I have been wondering if I should also prepare to become a government firefighter.”

(Participant 3)

As time passed, experienced nurses realized the limits to their vocational growth, and many male nurses considered transitioning to another career.

## 4. Discussion

Through examining the work and life experiences of mid-career male nurses, the qualitative results from the present study clarified the difficulties they experienced concerning long-term job retention.

The first theme cluster was “compliance with the workplace culture”, which consisted of “living in a vertical ward culture” and “paying your dues”. The female-centric, conservative, and hierarchical organizational structure observed in this study is consistent with that reported in some past research [25] and Korea’s clinical culture. We posit that a free communication system, in which male and female nurses within a nursing organization can cooperate and not be hindered by a strict vertical relationship, should be promoted within clinical settings.

Second, “being recognized as a member of the organization” comprised “adapting to a traditionally female environment” and “working smoothly”. Over time, male nurses achieved recognition and gained respect; however, this required trial and error, strenuous effort, and practice. This finding is consistent with that observed in previous research on male nurses with an average of 9.6 years of clinical experience, which found that a sense of visibility and representativeness made male nurses work harder to be a positive example [26].

Third, “new role burden” comprised “expectations concerning new work” and “taking initiative”. Participants were aware of the expectations of their department, and they expended the effort required to meet those expectations. This is similar to findings from several previous studies [18,21,25].

Fourth, “physical exhaustion” comprised “lack of sleep and chronic fatigue” and “burden of night shifts and a lack of physical fitness”. This is similar to previous findings that nurses of both genders experience exhaustion because of a lack of sleep due to irregular shift work and strenuous nursing duties [27,28,29]. To address these problems, administrative nursing departments should devise specific policies that include hiring nurses who exclusively work nights or limiting the number of consecutive night shifts that one must work.

Fifth, “strategy and support for survival” comprised “communicating with each other”, “support of male colleagues”, and “encouragement received from fellow and senior nurses”. It is not easy for some men to work in a workplace in which women form the majority; therefore, many male nurses find it difficult to form personal relationships with female nurses [30,31]. Previous studies [7,18,32] suggested the use of meetings and outside-work activities to build relationships with colleagues. However, the existence of other male nurses (including male nurses in other departments) was of great help to the nurses in this study. This is similar to the results of previous studies on male nurses’ adaptation to ward life [15,18].

Moreover, seeking support from senior nurses was a key component of maintaining and enduring clinical life. A past study showed that obtaining advice from seniors helps nurses experience a positive sense of identity [21]. Consequently, encouraging nurses to seek support from senior nurses is one strategy to promote personal relationships.

Sixth, “improved patient relationships” comprised “clinical performance is easier than before” and “positive perception toward male nurses”. Participants reported that, in forming a treatment relationship with patients, they became more adept, and the procedures became easier. At the beginning of the job, male nurses took a comparatively longer time to learn the job, lacked the ability to multitask, and were not skilled at forming a treatment relationship with patients as compared to female nurses [18]. However, the present findings suggest that male nurses seem to be able to address these issues with experience.

Moreover, with the increase in the number of male nurses in clinical settings, the representation of male nurses in the media is increasing. Consequently, the notion that male nurses are trustworthy and competent is being naturally ingrained in public perception [15].

Seventh, “growth limitations” comprised “familial financial burden” and “lack of role models”. Participants felt that their careers could not advance, which is consistent with past literature showing that male nurses are not commonly promoted to Head Nurse and that they experience less confidence and more anxiety with age [33,34,35]. We recommend that male nurses should be promoted to administrative and mentoring positions. Furthermore, some participants described themselves as pioneers in male nursing. This is consistent with the study by Meadus and Twomey [16] that noted the lack of role models for men in nursing. People working in other professions should be considered role models.

Eighth, “preparing for a better future” comprised “developing competence through self-reflection” and “considering changing job roles”. The present findings suggested that participants started thinking about and working toward their future with a self-directed attitude, which was contrary to prior results [18,25,36,37] that reported individuals express anxiety about the future if they continue through life without a particular plan. However, these findings seem similar in that participants had plan, and, therefore, were presumably less anxious about their future. To solve the problem of an insufficient number of qualified nurses, more male nurses need to be hired and retained [9]. Mid-career male nurses should not feel defeated regarding promotion; their career development requires ongoing effort to enable them to achieve their growth potential.

### Limitations

The fact that participants were nine mid-career male nurses recruited from general hospitals located in metropolitan cities is a limitation of this study. This is because it is difficult to generalize the results obtained to the clinical work and life of all male nurses. In addition, specific inclusion criteria may have limited the scope of the data acquired.

## 5. Conclusions

Male nurses are relatively few in number as compared to female nurses, which makes it more difficult for them to adapt to the strict hierarchical clinical culture and establish themselves as experienced nurses. A better working environment will be created if Korea’s nursing organizational culture voluntarily changes from a conservative and hierarchical culture to a cooperative organizational culture and system that is based on moral integrity within the organization. Additionally, male nurses worry about limited prospects for promotion and uncertainty regarding their future, and look for a “breakthrough”. Male nurses lack confidence concerning their competence as nurses and they are expected to actively demonstrate their expertise. Furthermore, nursing administrators must develop effective nursing policies to increase the long-term retention of clinical male nurses and to provide them an opportunity to contribute to the society.

### Implications

This study examined the strategies employed by mid-career male nurses in the nursing profession and their concerns about their vocational future. In addition to identifying ways to help prevent male nurses from leaving the clinical nursing profession, this study clarified the clinical working experience of male nurses, which can inform the development and implementation of nursing policies that support male nurses’ work adaptation.

## Figures and Tables

**Table 1:** Participants’ demographic characteristics.

Participant	Age (Years)	Religion	Working Department	Working Period (Years)	Spouse’s Job	Number of Children
1	38	Catholic	Emergency room	13	Nurse	2
2	34	Catholic	Operating room	9	Nurse’s aide	1
3	33	Christian	Psychiatric ward	7	Official	0
4	32	Buddhism	Operating room	7	Nurse	2
5	36	-	Emergency room	5	Nurse	1
6	37	Christian	Emergency room	8	Nurse	0
7	33	-	Emergency room	7	Housewife	1
8	30	Christian	Internal medicine ward	5	Nurse	0
9	31	-	outpatient cardiac center	5	Nurse	0

**Table 2:** Clinical work and life of mid-career male nurses.

Category	Theme Cluster	Theme
Limitations and adaptation to work performance	Compliance with the workplace culture	Living in a vertical ward culture Paying your dues
	Being recognized as a member of the organization	Adapting to a traditionally female environment Working smoothly
	New role burden	Expectations concerning new work Taking initiative
	Physical exhaustion	Lack of sleep and chronic fatigue Burden of night shifts and a lack of physical fitness
Interpersonal difficulties and coping	Strategy and support for survival	Communicating with each other Support of male colleagues Encouragement received from fellow and senior nurses
	Improved patient relationships	Easier clinical practice than ever before Positive perception toward male nurses
Facing reality and preparing for the future	Growth limitations	Familial financial burden Lack of role models
	Preparing for a better future	Developing competence through self-reflection Considering changing job roles

## Data Availability

The data presented in this study are available on request from the corresponding author. The data are not publicly available due to privacy.

## References

[1] Harding T., Jamieson I., Withington J., Hudson D., Dixon A. (2018). Attracting men to nursing: Is graduate entry an answer?. Nurse Educ. Pract..

[2] Korean Nurses Association Number of Male Nurses. http://www.koreanurse.or.kr/board/board_read.php?board_id=research&member_id=admin&exec=&no=39&category_no=2&step=0&tag=&sgroup=37&sfloat=&position=0&mode=&find=&search=.

[3] Korean Nurses Association Nursing Statistics. http://www.koreanurse.or.kr/.

[4] Yu M., Kang K.J., Yu S.J., Park M. (2017). Factors affecting retention intention of male nurses working health care institution in Korea. J. Korean Acad. Nurs. Adm..

[5] McKenna L., Vanderheide R., Brooks I. (2016). Is graduate entry education a solution to increasing numbers of men in nursing?. Nurse Educ. Pract..

[6] Yang C.I., Gau M.L., Shiau S.J., Hu W.H., Shih F.J. (2004). Professional career development for male nurses. J. Adv. Nurs..

[7] Ahn K.H., Seo J.M., Hwang S.K. (2009). Content analysis of male hospital nurses’ experiences. Korean J. Adult. Nurs..

[8] Yoon H., Choi J., Lee E., Lee H., Park M. (2013). Effects of decision making competency, nursing professionalism, and job satisfaction on turnover impulse among nurses. J. Korean Acad. Nurs. Adm..

[9] Zhang H., Tu J. (2020). The working experiences of male nurses in China: Implications for male nurse recruitment and retention. J. Nurs. Manag..

[10] Hollup O. (2014). The impact of gender, culture, and sexuality on Mauritian nursing: Nursing as a non-gendered occupational identity or masculine field? Qualitative study. Int. J. Nurs. Stud..

[11] Anker R. (1997). Theories of occupational segregation by sex: An overview. Int. Labour. Rev..

[12] Busch A. (2011). Determinants of Occupational Gender Segregation: Work Values and Gender Typical Occupational Preferences of Adolescents.

[13] Williams C.L. (2013). The glass escalator, revisited: Gender inequality in neoliberal times, SWS feminist lecturer. Gend. Soc..

[14] Muench U., Sindelar J., Busch S.H., Buerhaus P.I. (2015). Salary differences between male and female registered nurses in the United States. JAMA.

[15] Kim I., Shim H. (2016). Subjectivity on the job image of male nurses. Korean J. Soc. Sci. Study Subj..

[16] Meadus R.J., Twomey J.C. (2007). Men in nursing: Making the right choice. Can. Nurse.

[17] Park S., Kwon D.W., Kim D., Kim S.H. (2019). Influences of gender-related perceptions and experiences on nursing professionalism: A cross-sectional study. Nurs. Health Sci..

[18] Kim J.H., Park K.O., Kim J.K., Jun H.J., Lee J.H., Cho E.K., Kim S.H., Kim Y.H. (2016). An adaptation experience of male nurses at general nursing unit. J. Korean Acad. Nurs. Adm..

[19] Lee M.Y., Park M.R., Park M.M. (2014). Survey on the Status of Hospital Nursing Staff Placement.

[20] McMillian J., Morgan S.A., Ament P. (2006). Acceptance of male registered nurses by female registered nurses. J. Nurs. Sch..

[21] Kim S.H., Kim S.J., Kang H.K. (2003). Male nurses’ adaptation experience in clinical nursing settings. J. Korea Acad. Ind. Coop. Soc..

[22] Saunders B., Sim J., Kingstone T., Baker S., Waterfield J., Bartlam B., Burroughs H., Jinks C. (2018). Saturation in qualitative research: Exploring its conceptualization and operationalization. Qual. Quant..

[23] Colaizzi F. (1978). Existential-Phenomenological Alternatives for Psychology.

[24] Lincoln Y.S., Guba E.G. (1985). Naturalistic Inquiry.

[25] Lee K.J., Kim M. (2013). The relationship of gender role conflict and job satisfaction upon organizational commitment in male nurses. Korean J. Adult. Nurs..

[26] Rajacich D., Kane D., Williston C., Cameron S. (2013). If they do call you a nurse, it is always a “male nurse”: Experiences of men in the nursing profession. Nurs. Forum..

[27] Cho K.H., Yang H.K., Kim K.H., Cho Y.C. (2007). Fatigue symptoms and its related factors among clerical public officers. Korean J. Health Edu. Promot..

[28] Kim H., Lee J. (2017). Turnover experience of male nurses. J. Korean Acad. Nurs..

[29] Moon I., Lee Y. (2015). Factors influencing fatigue, physical health status and negative affectivity in shift-working nurses. J. Women’s Study.

[30] Park H.S., Ha J.H., Lee M.H. (2014). The relationship among gender-role identity, gender stereotype, job satisfaction and turnover intention of male nurses. J. Korea Acad. Ind. Coop. Soc..

[31] Shin J.H., Seo M.H., Lee M.I. (2016). Nursing jobs and gender in our age of convergence: Research on male nurses. J. Digit. Converg..

[32] Kim M. (2009). An exploratory study of masculinity in nursing. J. Korean Clin. Nurs. Res..

[33] Cho M.K., Kim C.G. (2016). The relationship among practice environment, organizational justice, and job satisfaction of male nurses. Korean J. Health Edu. Promot..

[34] Lee M.A. (2008). The relationship between the justice of compensation and the intention of turnover perceived by nurses. J. Korean Acad. Soc. Nurs. Educ..

[35] Son H.M., Koh M.H., Kim C.M., Moon J., Yi M. (2003). The male nurses’ experiences of adaptation in clinical setting. J. Korean Acad. Nurs..

[36] Ahn M.K., Lee M.H., Kim H.K., Jeong S.H. (2015). Job satisfaction, organizational commitment and turnover intention among male nurses. J. Korean Acad. Nurs. Adm..

[37] Yun H.J. (2016). A study on the adaption process of experienced male nurses. Asia Pac. J. Multimed. Serv. Converg. Art Humanit. Sociol..

